# Efficacy of Conservative Approaches on Pain Relief and Function in Patients With Rotator Cuff Calcific Tendinopathy: Which Is the Best Option? A Systematic Review and Network Meta‐Analysis

**DOI:** 10.1111/os.70175

**Published:** 2025-09-26

**Authors:** Lucrezia Moggio, Nicola Marotta, Alessandro de Sire, Giorgia Lucia Benedetto, Giorgio Gasparini, Antonio Ammendolia, Elvira Immacolata Parrotta, Michele Mercurio

**Affiliations:** ^1^ Rehabilitation Unit, Ospedale Degli Infermi Biella Italy; ^2^ Research Center on Musculoskeletal Health, MusculoSkeletalHealthatUMG Magna Graecia University Catanzaro Italy; ^3^ Department of Experimental and Clinical Medicine University of Catanzaro “Magna Graecia” Catanzaro Italy; ^4^ Department of Medical and Surgical Sciences University of Catanzaro “Magna Graecia” Catanzaro Italy; ^5^ Stem Cell Laboratory, Department of Medical and Surgical Sciences Magna Graecia University Catanzaro Italy; ^6^ Department of Medical and Surgical Sciences Magna Graecia University, Orthopaedic and Trauma Surgery, R. Dulbecco University Hospital Catanzaro Italy

**Keywords:** bursectomy, extracorporeal shockwaves therapy, rehabilitation, rotator cuff calcific tendinopathy, shoulder pain

## Abstract

**Objective:**

Rotator cuff calcific tendinopathy is a leading cause of nontraumatic shoulder pain, frequently leading to articular and functional impairments, depicting an adhesive capsulitis‐like clinical presentation. To date, there is a lack of evidence on the impact of conservative approaches, and no gold standard has been established for managing rotator cuff calcific tendinopathy. This systematic review aimed to identify the most effective conservative approach for reducing pain and improving function in rotator cuff calcific tendinopathy patients.

**Methods:**

PubMed, Scopus, and Cochrane Library databases were systematically searched from their inception until January 2, 2025, for English‐language randomized clinical trials including adults affected by rotator cuff calcific tendinopathy undergoing conservative treatment. Data extraction was performed independently by two reviewers using a customized data extraction form, with consensus reached by a third reviewer. A network meta‐analysis was subsequently carried out to compare the efficacy of different interventions. The risk of bias within the included randomized clinical trials was assessed using Version 2 of the Cochrane risk‐of‐bias tool for randomized trials. The study has been registered with PROSPERO, registration number CRD420250650833.

**Results:**

Nineteen articles were included. This study identified 1160 subjects affected by rotator cuff calcific tendinopathy. A pairwise comparison through a network meta‐analysis indicated that platelet‐rich plasma exhibited the highest probability (85%) of improving shoulder function, followed by disodium ethylenediamine tetra‐acetic acid at 75%, aspiration techniques at 65%, and extracorporeal shockwave therapy at 57%. Regarding pain reduction, disodium ethylenediamine tetra‐acetic acid showed the highest probability (66%), followed by kinesiotaping and needle aspiration, both at 61%.

**Conclusion:**

This systematic review and network meta‐analysis identified several interventional techniques, including platelet‐rich plasma and disodium ethylenediamine tetra‐acetic acid injections, extracorporeal shockwave therapy, and needle aspiration, as more effective strategies for reducing pain and improving function in subjects affected by rotator cuff calcific tendinopathy.

**Level of Evidence:**

I (systematic review of Level‐I randomized controlled studies).

## Graphical abstract

Effect of each approach via SUCRA ranking analysis (0%–100%) on Constance‐Murley score (CMS) (a) and perceived pain (b). EDTA: ethylene diamine tetra‐acetic acid; ESWTh: extracorporeal shockwaves therapy high; ESWTl: extracorporeal shockwaves therapy low; ESWTm: extracorporeal shockwaves therapy medium; PRP: platelets‐rich plasma; US: ultrasound.

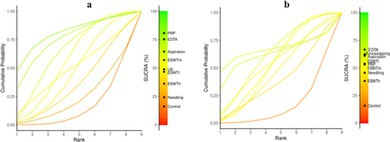

**Abbreviations**
CMS: Constant–Murley shoulder assessment
EDTA: disodium ethylenediamine tetra‐acetic acid
EFD: energy flux density
ESWT: extracorporeal shock wave therapy
KT: kinesiotaping
NRS: numerical rating scale
PICO: participants, intervention, control, outcomes
PRISMA: preferred reporting items for systematic reviews and meta‐analyses
PRP: platelet‐rich plasma
RCCT: rotator cuff calcific tendinopathy
RCTs: randomized controlled trials
rESWT: radial ESWT
SUCRA: surfaces under the cumulative ranking curves
US: ultrasound
VAS: visual analog scale

## 1 Introduction

Rotator cuff calcific tendinopathy (RCCT) is a common cause of non‐traumatic shoulder pain. This condition involves the deposition of carbonate hydroxyapatite crystals within the rotator cuff tendons, mainly affecting individuals aged 30 to 60 years. Women are disproportionately affected, with a prevalence about 1.5 times higher than men. Notably, bilateral deposits are seen in 10%–20% of patients [1]. The supraspinatus tendon is the most commonly affected site, followed by the inferior part of the infraspinatus tendon and the pre‐insertional area of the subscapularis tendon [2]. Pinter first described RCCT in 1907, and subsequent studies have clarified its clinical and radiologic features. Gartner's classification remains the most widely used framework for categorizing this condition [3]. Gartner and Heyer described a two‐phase disease process: the initial chronic phase features a dense, well‐defined calcific deposit (type I), while the acute phase involves spontaneous resolution, leading to a translucent, cloudy appearance without clear borders (type III). In some cases, the specific X‐ray morphology of the calcific deposit cannot be identified (type II) [4]. The authors outlined a comprehensive three‐phase progression: pre‐calcific, calcific (which includes formative, resting, and resorptive subphases), and post‐calcific stages. During these stages, patients often experience severe, disabling pain that may not be related to movement. These symptoms frequently accompany joint and functional impairments, resembling adhesive capsulitis (frozen shoulder) [5, 6]. Various treatment options, from conservative therapies to surgery, have been proposed for RCCT.

The algorithm for managing RCCT begins with a non‐invasive approach, involving modifications to physical and/or work activities, administration of anti‐inflammatory medications, and physiotherapy. In this context, several minimally invasive treatments and rehabilitative strategies can be considered for patients with shoulder pain conditions [7, 8, 9, 10, 11, 12].

In this context, ultrasound (US) therapy is often used to treat painful musculoskeletal disorders [13]. However, its use remains mostly empirical, based on reported biophysical effects within tissues and anecdotal evidence rather than strong, evidence‐based research [14]. Several studies [15, 16, 17] have shown that ultrasound therapy can provide short‐term relief from symptoms; however, how ultrasound facilitates the resorption of calcium deposits is still unclear [18]. Kinesiotaping is frequently used in preventing and treating sports‐related injuries due to its four main physiological effects: facilitating or inhibiting muscle function, improving blood and lymphatic circulation, providing pain relief, and correcting abnormal joint alignment [18, 19]. Disodium ethylenediamine tetra‐acetic acid (EDTA), a heavy metal and mineral chelator, has gained popularity recently as a treatment for calcific tendinitis of the shoulder. It is usually administered through ionophoresis or mesotherapy, showing favorable results [20, 21]. Platelet‐rich plasma (PRP) has also become a common treatment for tendinopathies [22], with promising results in conditions such as lateral epicondylitis and patellar tendinopathy [23, 24]. Notably, compared to corticosteroid injections, PRP appears to provide better short‐term outcomes in patients with partial‐thickness rotator cuff tears [25]. Sonographic‐guided needle puncture directly targeting the calcium deposit in the shoulder has been confirmed as an effective and minimally invasive option [26]. This procedure involves performing multiple percutaneous punctures at each deposit site without aspiration [27, 28, 29, 30]. Some studies suggest that complete removal of the deposit may not be necessary for significant clinical improvement [31]. Similar to needling, needle aspiration (also called barbotage or lavage) of the calcific deposit is widely used in managing RCCT due to its effectiveness in reducing pain and improving function [22, 29, 32, 33]. However, this invasive procedure requires specialized skills and equipment and can be time‐consuming, often causing discomfort during and after the intervention [34]. Extracorporeal shock wave therapy (ESWT) was first introduced for musculoskeletal disorders by Valchanou and Michailov [35], initially to treat delayed nonunion of fractures and pseudoarthrosis. The mechanical effect of ESWT (cavitation effect) promotes calcium deposit reabsorption by increasing blood flow and oxygen pressure around the deposit, and by inducing phagocytosis of the calcium deposit through neovascularization, inflammatory response, and leukocyte chemotaxis, [30, 36, 37]. Several studies have explored the role of ESWT in treating RCCT, with mixed results [18, 27, 28, 30, 33, 38, 39, 40, 41, 42, 43, 44, 45]. Additionally, other treatments such as prolotherapy [46 p. 2], corticosteroid injections in the subacromial space [47, 48, 49, 50], electroacupuncture [51], and acetic acid iontophoresis [52, 53] are often used alongside other techniques. For refractory cases, surgery involving debridement and arthroscopic tendon repair may be necessary [37, 54]. In 2018, Chianca et al. [55] reviewed different therapeutic approaches for RCCT; however, this review was not systematic and offers only a general overview of the main methods reported in the literature.

Therefore, to the best of our knowledge, there is still a lack of evidence on the impact of conservative approaches, and no standard of care has been established for the management of patients affected by RCCT. Thus, by the present systematic review and meta‐analysis, we aimed to investigate the most effective conservative approach to reduce pain and improve function in patients affected by RCCT. Specifically, the objectives of this study were: (1) to compare the efficacy of different conservative treatments in terms of pain relief and functional recovery; (2) to rank these interventions according to their probability of effectiveness; and (3) to provide evidence‐based guidance for clinical decision‐making in the conservative management of RCCT.

## 2 Methods

### 2.1 Search Strategy

PubMed, Scopus, and Cochrane Library databases were systematically searched for English‐language articles published from their inception to January 2, 2024, utilizing specific thesaurus terms (Table 1).

**TABLE 1:** Search strategy.

PubMed (“shoulder” OR “rotator cuff”) AND (“tendonitis” OR “tendinitis” OR “tendinopathy”) AND (“calcific” OR “calcification” OR “deposit”) AND (“rehabilitation” OR “exercise” OR “physical therapy” OR “iontophoresis” OR “corticosteroid” OR “needling” OR “ultrasound‐guided” OR “lavage” OR “platelet‐rich plasma” OR “shock waves” OR “surgical treatments” OR “arthroscopy”)
Scopus TITLE‐ABS‐KEY(((“shoulder” OR “rotator cuff”) AND (“tendonitis” OR “tendinitis” OR “tendinopathy”) AND (“calcific” OR “calcification” OR “deposit”) AND (“rehabilitation” OR “exercise” OR “physical therapy” OR “iontophoresis” OR “corticosteroid” OR “needling” OR “ultrasound‐guided” OR “lavage” OR “platelet‐rich plasma” OR “shock waves” OR “surgical treatments” OR “arthroscopy”)))
Cochrane library ((“shoulder” OR “rotator cuff”) AND (“tendonitis” OR “tendinitis” OR “tendinopathy”) AND (“calcific” OR “calcification” OR “deposit”) AND (“rehabilitation” OR “exercise” OR “physical therapy” OR “iontophoresis” OR “corticosteroid” OR “needling” OR “ultrasound‐guided” OR “lavage” OR “platelet‐rich plasma” OR “shock waves” OR “surgical treatments” OR “arthroscopy”))

This systematic review with meta‐analysis was conducted following the guidelines outlined by the preferred reporting items for systematic reviews and meta‐analysis (PRISMA) statement and the Cochrane Handbook for Systematic Reviews of Interventions. The protocol for this systematic review is registered with the International Prospective Register of Systematic Reviews (PROSPERO) under registration number: CRD420250650833.

### 2.2 Selection Criteria

After duplicate removal, two reviewers independently screened all papers for eligibility. In case of disagreement, a consultation with a third reviewer allowed consensus. Articles were considered eligible if they addressed the questions outlined by the following PICO model:

(P) Participants: patients affected by RCCT for ≥ 3 months, with imaging‐confirmed calcific deposits (via ultrasound and/or radiography) Gärtner type I or II calcifications.

(I) Intervention: conservative and rehabilitative treatment aimed at reducing pain and improving function in RCC.

(C) Comparator: rehabilitative approaches considered as interventions, including placebo, sham treatment, or conventional rehabilitation.

(O) Outcome measure: pain intensity assessed using the visual analog scale (VAS) or numerical rating scale (NRS), and functional outcomes using Constant–Murley Shoulder Assessment (CMS).

Specifically, the interventions evaluated in the included studies were categorized based on treatment modality and specific parameters. ESWT interventions were classified according to energy levels and application protocols. To stratify the results, we divided the treatments delivered into high (EFD > 0.3 mJ/mm^2^), medium (EFD > 0.2 mJ/mm^2^ but < 0.3 mJ/mm^2^), and low (EFD < 0.2 mJ/mm^2^) EFD. Treatment sessions, frequency, and total number of shocks were also considered when reported. Rehabilitative treatments included physiotherapy, exercise therapy, manual therapy, and modalities aimed at improving shoulder function and reducing pain. The specific type and duration of rehabilitation protocols were extracted and grouped accordingly. Other interventions, such as corticosteroid injections, ultrasound therapy, and placebo/sham treatments, were also classified as distinct categories. This classification scheme was used to synthesize the evidence and conduct the network meta‐analysis, allowing for meaningful comparisons across treatment types.

We included randomized controlled trials (RCTs) with two groups (study group and control group) reporting the data of > 10 treated cases. We excluded: (1) studies involving children or growing subjects; (2) studies focusing on treatment for other complications related to RCCT (e.g., tendons rupture); (3) cross‐over study design; (4) studies written in a language different from English; (5) full‐text unavailability (i.e., posters and conference abstracts); (6) studies involving animals.

We included only RCTs that assessed the CMS score to minimize heterogeneity across studies and enhance the comparability of functional outcomes in our network meta‐analysis.

### 2.3 Data Extraction

Two reviewers independently extracted relevant data from the included studies using a customized data extraction template on a Microsoft Excel sheet. In case of disagreement, a third reviewer facilitated consensus. The following data were extracted: (1) first author; (2) publication year; (3) nationality; (4) age and sex of study participants; (5) affected side; (6) dominant hand; (7) smoking behavior; (8) thyroid disease or diabetes mellitus; (9) therapeutic approach performed; (10) comparator; (11) population and the number of patients included; (12) Pain and functional outcome measures; (13) Main findings. The selected studies have been synthesized by describing extracted data.

### 2.4 Outcome Measures

Studies with incomplete outcome data were assessed, and where possible, authors were contacted for clarification. Missing data were handled using available‐case analysis in the meta‐analyses.

The primary outcomes were defined as pain intensity (measured by VAS or NRS) and shoulder function (measured by Constant‐Murley Score). Secondary outcomes, when reported, included quality of life measures, adverse events, and patient satisfaction.

### 2.5 Quality Assessment

To assess the methodological quality of the included studies, specifically regarding the randomization process, deviations from intended interventions, missing outcome data, data measurement, and reported results, two independent reviewers assessed RCT's study quality using the Cochrane risk‐of‐bias tool Rob2 [56]; this tool allowed us to analyze the randomization process, deviations from intended interventions, missing outcome data, measurement of outcomes, and selection of reported results. Each domain was rated as low, some concerns, or high risk of bias. The trials were graded as unclear, high, or low risk of bias. We excluded studies with imputed data to evaluate the robustness of the results. We assessed publication bias via Egger's test where applicable.

### 2.6 Statistical Analysis

All data were reported with one‐decimal accuracy. The mean, standard deviation, and range were reported for continuous variables, and the count was reported for categorical variables. Statistical analysis was performed using RevMan 5.4 and R 4.3.0 (R foundation, Wien, Austria). Heterogeneity among comparisons was assessed via I2 tests; thus, an I2% > 50% determined significant heterogeneity across the manuscripts. A random‐effects model was employed to calculate pooled estimates with 95% confidence intervals (95% CIs). Following methodologies established in prior reviews [57, 58, 59], a network meta‐analysis was performed, integrating results from each RCT to estimate an overall effect by connecting direct head‐to‐head interventions alongside indirect comparisons from different trials. This approach allowed for the computation of surfaces under the cumulative ranking curves (SUCRA) scores, ranking each treatment based on its effectiveness. The SUCRA score provides a probabilistic ranking of treatments based on their relative effectiveness, but small differences in SUCRA values do not necessarily imply clinically meaningful differences. Moreover, treatment rankings were interpreted alongside effect sizes and confidence intervals to assess clinical relevance.

Each intervention was assigned a SUCRA score ranging from 0% to 100%. Ultimately, we generated a ranking of the included interventions based on their SUCRA score, incorporating both direct head‐to‐head and indirect comparisons within the meta‐analysis network. The statistical weight for each class and intervention was estimated using the Markov chain Monte Carlo; the software ran two chains with different initial values simultaneously to evaluate convergence, using the Gelman–Rubin diagnostic trace plots, and comparing direct and indirect estimates in each triangular loop. This analysis method minimizes bias and indirectly evaluates the best treatment among multi‐arm studies. Additionally, a Node‐splitting model was implemented to assess whether any inconsistencies in treatment comparisons relaxed the consistency assumption for one comparison while maintaining it across the entire network. A *p* value of less than 0.05 was considered indicative of statistical significance.

## 3 Results

### 3.1 Study Characteristics

A total of 1136 articles were identified across all searched databases using the applied research strategy. Upon removal of duplicates, 744 papers were reviewed and filtered for relevance based on their titles and abstracts, which led to the exclusion of 680 articles. This process resulted in the identification of 64 full‐text articles, which were retrieved for detailed evaluation (see Table 2 for further details on the excluded studies).

**TABLE 2:** Reasons for article exclusion by the present systematic review.

Articles excluded after title and abstract screening phase (*n* = 643)*	
Study design	542 (84.30%)
Not population of interest	79 (12.28%)
No intervention of interest	2 (0.31%)
Not comparison of interest	0 (0%)
Not outcome of interest	10 (1.55%)
No abstract available	10 (1.55%)

Articles excluded after full‐text screening phase (*n* = 45)	
Study design	17 (30.7%)
Not population of interest	7 (15.55%)
Not intervention of interest	5 (11.11%)
Not comparison of interest	0 (0%)
Not outcome of interest	16 (35.55%)

*Note*: The exclusion of the articles followed the PICO model defined in the Methods Section. Data are expressed as counts (percentages). * = Papers were excluded also for more than one reason during the title and abstract screening phase and the full‐text screening phase.

**FIGURE 1:**
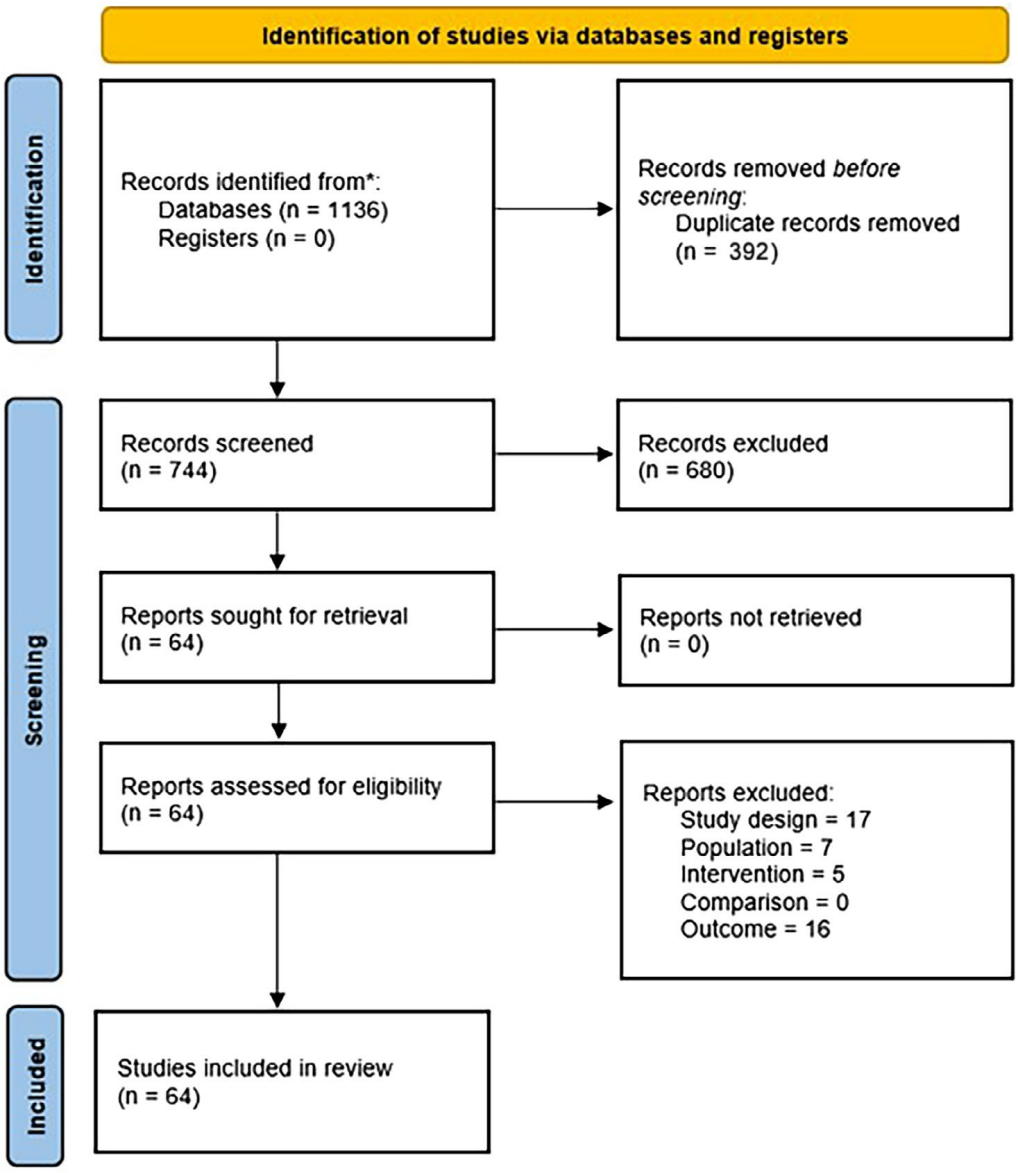
PRISMA flow chart.

Therefore, 19 RCTs [18, 21, 22, 27, 28, 29, 30, 33, 38, 39, 40, 41, 42, 43, 44, 45, 60, 61, 62] were included in our systematic review (see Figure 1 for PRISMA flow diagram). The included studies have been published from 1999 [60] to 2022 [45]. Fourteen (73,6%) were conducted in Europe (5 from Austria [28, 39, 41, 42, 60], 4 from the Netherlands [22, 27, 33, 61], 3 from Italy [18, 21, 43], 1 from Germany [38], and 1 in France [40]), and 5 (26,3%) in Asia (2 in South Korea [30, 62], 1 in Turkey [44], 1 in Pakistan [45], and 1 in China [29]). We analyzed 1160 subjects affected by RCCT, 575 of whom were included in the control group. Sample sizes ranged from 14 [33, 42] to 41 [27, 29] in the experimental and control groups, with a mean age ranging from 46 (2) years [21, 40] to 57 (16) years [43], whereas the time from the onset of symptoms to treatment was variable from 6 months to 3 years, including subjects in the subacute or chronic phase. Table 3 summarizes the main characteristics of the RCTs included in our systematic review.

**TABLE 3:** Main characteristics of the randomized controlled trials included in the present systematic review.

Article	Nationality	Study group	Control group	Intervention	Comparison	Drop‐out	Outcome measure	Follow‐up	Main findings
Ebenbichler et al. NEJM 1999 [60]	Austria	*n* = 32; −M/−F Age: 49 ± 11 years Type of lesion: I 27/II 5 Affected shoulder: 13 L/19 R Onset (months): 8 (4–20)	*n* = 29; − M/−F Age: 54 ± 10 years Type of lesion: I 20/II 9 Affected shoulder: 15 L/14 R Onset (months): 8 (4–19)	US pulsed‐mode 15′ per session to the area over the calcification (frequency 0.89 MHz, intensity 2.5 W/cm^2^) with a transducer of 5 cm^2^; the first 15 of 24 treatments were given daily (5/week) for 3 weeks, and the remaining 9 were given 3/week for 3 weeks.	Sham treatment (with ultrasonic generator not turned on)	9	CMS	baseline, 6 weeks, and 9 months after treatment	At 6 weeks, the experimental group showed a greater decrease in pain, and quality of life greater improvements compared to the sham group; at 9 months, the differences between the groups were no longer significant.
Perlick et al. J Orthop Sci 2003 [38]	Germany	80 patients (36 men and 44 women) with a mean age of 48.4 years (range 38–64 years). The mean duration of symptoms was 32 months. *n* = 40 each group		ESWT 2000 impulses (EFD 0.23 mJ/mm2), 2 sessions, 3 weeks apart. .	ESWT 2000 impulses (EFD 0.42 mJ/mm2), 2 sessions, 3 weeks apart.	0	CMS	baseline, 3 and 12 months after treatment	After 1 year, the CMS increased from 46 to 68 at 0.23 mJ/mm2 and from 48 to 73 points at 0.42 mJ/mm2.
Pleiner et al. Wien Klin Wochenschr 2004 [39]	Austria	*n* = 23; 8 M/15 F Age: 54 ± 11 years Type of lesion: ‐ Affected Shoulder: 18 L/8 R Onset (months): at least 6 months	*n* = 20; 4 M/16 F Age: 50 ± 8 years Type of lesion: ‐ Affected Shoulder: 16 L/4 R Onset (months): at least 6 months	ESWT 2000 impulses (EFD 0.28 mJ/mm2), 2 sessions, 2 weeks apart.	ESWT 2000 impulses (EFD < 0.07 mJ/mm2) in 2 sessions, 2 weeks apart.	10	CMS and VAS	Baseline, and 1 week, 3 months, and 7 months after treatment	Improvement in CMS was significantly higher in the treatment group at all follow‐up visits (*p* < 0.05). significant improvement in the treatment group compared with the control group at the 1‐week follow‐up (*p* < 0.05).
Krasny et al. J Bone Joint Surg 2005 [28]	Austria	*n* = 40; 24 M/16 F Age: 47.3 (32.5–67.3) years Type of lesion: 18 I/22 II Affected Shoulder: 17 L/23 R Onset (months): 36.3 (13–96)	*n* = 40; 15 M/25 F Age: 49.4 (32.4 to 63.5) years Type of lesion: 21 I/19 II Affected Shoulder: 13 L/27 R Onset (months): 30.5 (12.0–60.0)	US‐guided needling + ESWT (2500 impulses, EDF 0.36 mJ/mm2), 1 session.	ESWT (2500 impulses, EDF 0.36 mJ/mm2), 1 session.	0	CMS	Baseline and 4 months after treatment	Both groups had significant improvement in CMS. Significantly better clinical and radiological results were obtained in group I than in group II.
Albert et al. J Bone Joint Surg 2007 [40]	France	*n* = 40; 9 M/31 F Age: 46.6 (31–64) years Type of lesion: ‐ Affected Shoulder: 10 L/30 R Onset (months): 41.2 (6–120)	*n* = 45; 27 M/18 F Age: 47.5 (32 to 69) years Type of lesion: ‐ Affected Shoulder: 27 L/18 R Onset (months): 36.4 (7–160)	ESWT 2500 impulses (EDF 0.45 mJ/mm2), 2 sessions, 2 weeks apart.	ESWT 2500 impulses (EDF gradually increased from 0.02 mJ/mm2 to 0.06 mJ/mm2).	0	CMS and VAS	At baseline and around 110 days after treatment	The improvement from the baseline was significant in the high‐energy group, with a mean gain of 12.5 (−20.7 to 47.5) points (*p* < 0.0001). The improvement was not significant in the low‐energy group.
Sabeti et al. Wien Klin Wochenschr 2007 [41]	Austria	44 patients (14 men and 30 women) Group I: 49.38 (±8.37) years, 21 patients Group II: 53.57 (±8.80) years, 23 patients		X‐ray‐assisted ESWT 1000 impulses (EDF 0.08 mJ/mm2), 3 sessions at weekly intervals.	X‐ray‐assisted ESWT 2000 impulses (EDF 0.2 mJ/mm^2^), 2 sessions at weekly intervals.	3	CMS and VAS	at baseline and 12 weeks after treatment	Both groups improved significantly (*p* < 0.0001) in the CMS and in the VAS. The statistics within‐ groups were not significantly different.
Zhu et al. Adv Ther 2008 [29]	China	*n* = 41; 26 M/15 F Age: 52.3 (44–71) years Type of lesion: ‐ Affected Shoulder: 12 L/29 R Onset (months): 11 (6–15)	*n* = 40; 24 M/16 F Age: 46.6 (31–64) years Type of lesion: ‐ Affected Shoulder: 15 L/25 R Onset (months): 10 (5–14)	US‐guided needling with a 16‐gauge needle, positioned in the calcification and gently rotated; lavage using a 10 mL syringe filled with sterile water for injection.	Needling with a 16‐gauge needle.	0	VAS	at baseline and 1, 2, 3, 6, 12, 24, and 36 weeks after treatment	In both groups, VAS significantly decreased over the 36 weeks following treatment (*p* < 0.05). Overall, the majority of the VAS scores were not statistically different between groups.
Cacchio et al. Arthritis & Rheumatism 2009 [21]	Italy	*n* = 40; 22 M/18 F Age: 46.12 ± 1.98 years Type of lesion: 8 I/32 II Affected Shoulder: 8 L/32 R Onset (months): 11 ± 3.5	*n* = 40; 22 M/18 F Age: 46.12 ± 1.98 years Type of lesion: 6 I/34 II Affected Shoulder: 10 L/30 R Onset (months): 12 ± 5.7	Mesotherapy with 1 mL of disodium EDTA, 1 mL of 1% procaine, and 3 mL of injectable water + US pulsed‐mode 15′ administered using a mixture of disodium EDTA and aquasonic gel, 5/week for 3 weeks.	Sham treatment (the ultrasonic generator not turned on, and the disodium EDTA not present in the mixture for mesotherapy and in the aquasonic gel for ultrasounds).	0	CMS and VAS	At baseline, 1 week, and 1 year after treatment	The study group displayed improvement in all of the parameters analyzed after treatment, and at the 1‐year follow‐up.
Farr et al. Knee Surg Sports Traumatol Arthrosc 2011 [42]	Austria	*n* = 15 Age: 49.7 ± 9.0 years	*n* = 15 Age: 48.6 ± 7.3 years	ESWT 3200 impulses (EDF 0.3 mJ/mm2), 1 session.	ESWT 1600 impulses (EDF 0.2 mJ/mm2), 2 sessions at weekly intervals.	3	CMS and VAS	At baseline, and 6 and 12 weeks after treatment	In both groups, a significant reduction in pain during stress and improvement of function was observed. In contrast, no significant reduction in pain during rest was observed.
Ioppolo et al. Physical Therapy 2012 [43]	Italy	*n* = 23; 8 M/15 F Age: 57.09 ± 16.40 years Type of lesion: 5 I/18 II Affected Shoulder: 7 L/16 R Onset (months): 6.95 ± 1.06	*n* = 23; 7 M/16 F Age: 51.65 ± 12.23 years Type of lesion: 6 I/17 II Affected Shoulder: 9 L/14 R Onset (months): 7.22 ± 1.2	ESWT 2400 impulses (EDF 0.20 mJ/mm2), 1/week for 4 weeks.	ESWT 2400 impulses (EDF 0.10 mJ/mm2), 1/week for 4 weeks.	10	CMS and VAS	baseline, and 3 and 6 months after treatment (CMS); at baseline, and 3 and 6 months after treatment (VAS)	Significant clinical improvement based on mean CMS was observed after 6 months in group A (*X* = 79.43, SD = 10.33) compared to group B (*X* = 57.91, SD = 6.53). Likewise, after 6 months, a significant decrease in VAS scores was found in group A (*X* = 2.09, SD = 1.54) compared to group B (*X* = 5.36, SD = 0.78).
de Witte et al. Am J Sports Med 2013 [61]	Netherlands	n = 23; 11 M/12 FAge: 53.7 ± 7.3 yearsType of lesion: ‐Affected Shoulder: 7 L/16 ROnset (months): —	n = 25; 12 M/13 FAge: 50.4 ± 7.2 yearsType of lesion: ‐Affected Shoulder: 6 L/19 ROnset (months): —	US‐guided needling with a 18‐gauge needle, positioned in the calcification and gently rotated; lavage using a 10 mL syringe filled with sterile water for injection +5 mL of bupivacaine and 1 mL of methyprednisolone 40 mg/mL in the subacromial bursa.	5 mL of bupivacaine and 1 mL of methyprednisolone 40 mg/mL in the subacromial bursa	0	CMS	At baseline and 6 weeks and 3, 6, and 12 months after treatment	At 1‐year follow‐up, the mean CS in group 1 was 86.0 (95% CI, 80.3–91.6) versus 73.9 (95% CI, 67.7–80.1) in group 2 (*p* = 0.005). Follow‐up scores were significantly influenced by baseline scores.
Kim et al. J Shoulder Elbow Surg 2014 [30]	South Korea	*n* = 25; 2 M/23 F Age: 53.9 (45–76)years Type of lesion: ‐ Affected Shoulder: ‐ Onset (months): 21.2	*n* = 29; 3 M/26 F Age: 57.4 (47–78) years Type of lesion: ‐ Affected Shoulder: ‐ Onset (months): 25.2	US‐guided needling with an 18‐gauge needle +1 mL of methyprednisolone 40 mg/mL in the subacromial bursa.	ESWT 1000 impulses (EDF 0.36 mJ/mm2), 3 sessions, 1 week apart.	8	VAS	at baseline and 6 weeks, 12 weeks, 6 months, 12 months after treatment	There were also significant improvements in clinical outcomes in both groups after treatment (*p* < 0.05).
Kim and Kwak J Phys Ther Sci 2016 [62]	South Korea	n = 18; 7 M/11 FAge: 50.2 ± 5.6 yearsType of lesion: ‐Affected Shoulder: 16 D/2ndOnset (months): —	n = 25; 4 M/12 FAge: 53.0 ± 4.6 yearsType of lesion: ‐Affected Shoulder: 16 D/2ndOnset (months): —	TENS 15‐min (100 Hz with a density of 20–30 mA) + 5‐min US therapy (1 W/cm2), 3 times/week for 12 weeks + ESWT 240 impulses (EDF 0.14 mJ/mm2), 3/week until 6 weeks, but not from 6 to 12 weeks.	TENS 15‐min (100 Hz with a density of 20–30 mA) + 5‐min US therapy (1 W/cm2), 3/week for 12 weeks.	6	CMS	at baseline, and 2, 6, and 12 weeks after treatment	The CMS showed a significant difference in the interaction of the groups according to measurement period (*p* < 0.05). The treatment group showed a more significant decrease in pain at 2, 6, and 12 weeks compared to the control group (*p* < 0.05).
De Boer et al. J Orthop 2017 [33]	Netherlands	*n* = 11; 6 M/5 F Age: 53 [95% CI 50–57] years Type of lesion: ‐ Affected Shoulder: 14 D/6nd Onset (months): 6 months at least	*n* = 14; 7 M/7 F Age: 53 [95% CI 48–58] years Type of lesion: ‐ Affected Shoulder: 14 D/6nd Onset (months): 6 months at least	Lavage with 2 hollow 18 gauge needles using a 10 mL syringe filled with sterile water flushed through both needle portals.	rESWT 500 impulses (EFD 0.10 mJ/mm), 4 sessions, one week apart.	6	CMS and NRS	at baseline and 6 weeks and 1 year after treatment	Lavage decreased deposit more than rESWT (*p* = 0.029). After 6 weeks, CMS and NRS improved more in lavage. After 1 year, there was no significant difference in NRS (*p* = 0.45).
Frassanito et al. Eur J Phys Rehabil Med 2018 [18]	Italy	n = 21; 7 M/14 FAge: 54.1 ± 10.3 yearsType of lesion: ‐Affected Shoulder: 9 L/12 ROnset (months): —	n = 21; 9 M/12 FAge: 48.7 ± 11.9 yearsType of lesion: ‐Affected Shoulder: 11 L/12 ROnset (months): —	ESWT 1800 impulses (EDF 0.07–0.15 mJ/mm2), 3 sessions, 1/week for 3 consecutive weeks + kinesiotaping on deltoid and supraspinatus muscles.	ESWT 1800 impulses (EDF 0.07–0.15 mJ/mm2), 3 sessions, 1/week for 3 consecutive weeks.	8	VAS	at baseline and 1, 4, and 12 weeks after treatment	Both groups showed significant improvement in all outcome measures. At the T1, improvement was significantly better in ESWT+kinesiotaping than ESWT on VAS (*p* = 0.007). Successive improvements at T2 vs. T1 and T3 vs. T2 did not differ significantly between the groups. At the end of follow‐up, ESWT+kinesiotaping still showed significantly greater improvement than ESWT on VAS (*p* = 0.02).
Duymaz and Sindel Arch Rheumatol 2019 [44]	Turkey	*n* = 40 Age: 54.33 ± 9.88 years	*n* = 40 Age: 51.31 ± 8.86 years	ESWT 1500 impulses (EDF 0.28 mJ/mm2), 1 session a week for 4 weeks + conventional rehabilitation with US, TENS, shoulder joint ROM and stretching exercises, and ice application, 5/week for 4 weeks.	Conventional rehabilitation with US, TENS, shoulder joint ROM and stretching exercises, and ice application, 5/week for 4 weeks.	0	VAS	at baseline and after treatment	All parameters in both groups improved significantly; patients in the ESWT group had a statistically significant improvement in pain, (*p* < 0.001).
Louwerens et al. Arthoscopy 2020 [27]	Netherlands	*n* = 41; 14 M/27 F Age: 51.6 (9.4) years Type of lesion: 13 I/28 II Affected Shoulder: ‐ Onset (years): 3.4 (3.0)	*n* = 41; 15 M/26 F Age: 52.7 (8.7) years Type of lesion: 21 I/20 II Affected Shoulder: ‐ Onset (years): 3.0 (3.0)	ESWT 2000 pulses (EDF 0.35 mJ/mm2), 4 sessions at weekly intervals.	US‐guided needling in a single session combined with a corticosteroid US‐guided subacromial bursa injection.	1	CMS and VAS	at baseline, and 6 weeks, 3, 6 and 12 months after treatment	At 1‐year, the needling group showed similar results as the ESWT group with regard to CMS (20.9 versus 15.7; *p* = 0.23), and VAS for pain (−3.9 and −2.6; *p* = 0.12).
Oudelaar et al. Am J Sports Med 2021 [22]	Netherlands	n = 39; 16 M/23 FAge: 48.5 ± 6.3Type of lesion: 9 I/30 IIAffected Shoulder: 14 L/24 ROnset (months): —	n = 41; 14 M/27 FAge: 48.8 ± 5.8 yearsType of lesion: 13 I/28 IIAffected Shoulder: ‐Onset (months): —	Lavage with 20–21 gauge needle using a 10 mL syringe filled with sterile water +4 mL of bupivacaine 2.5 mg/mL, and 1 mL of triamcinolone acetonide 40 mg/mL injection in the subacromial bursa.	Lavage with 20–21 gauge needle using a 10 mL syringe filled with sterile water + PRP injection in and around the affected rotator cuff tendon.	10	NRS and CMS	At baseline, and 6 weeks, and 3, 6, 12, and 24 months after treatment	Both groups showed improvement of clinical scores at the 2‐year follow‐up (*p* < 0.001 for all clinical scores). NACD+PRP was found to be noninferior to NACD+corticosteroids with regard to the mean decrease of NRS scores (4.34 vs. 3.56; *p* = 0.003)
Fatima et al. BioMed Research International 2022 [45]	Pakistan	*n* = 21 Age: 48.7 ± 6.74	*n* = 21 Age: 49.8 ± 7.54	ESWT 2000 impulses (EDF 0.32 mJ/mm2), 12 sessions for the first 6 weeks (2 sessions/week) + standard physical therapy, 12 sessions for 6 weeks (2 sessions/week).	Standard physical therapy, 12 sessions for 6 weeks (2 sessions/week).	2	NPRS and CMS	at baseline, and after 6 and 12 weeks after treatment	There were significant differences regarding NPRS and CMS between the two groups, at baseline and 6th and 12th weeks after intervention (*p* < 0.05). Within‐group differences also showed statistically significant results after treatment (all *p* < 0.05)

### 3.2 US

Ebenbichler et al. [60] analyzed 70 shoulders (63 patients) affected by RCCT undergoing 15‐min sessions of either pulsed US (frequency 0.89 MHz; intensity 2.5 W per square centimeter; pulsed mode, 1:4) or an indistinguishable sham treatment to the area over the calcification; they found that 75% of shoulders in the US‐treatment group presented a normal CMS score against only 34% of shoulders in the sham‐treatment group at the end of therapy. Moreover, the improvement of the CMS pain section was significantly better in the treatment group than in the sham one (6.4 vs. 1.6). Furthermore, US is often utilized in conjunction with other techniques [21, 62].

### 3.3 Kinesiotaping

Frassanito et al. [18] investigated the efficacy of kinesiotaping (KT) applied on deltoid and supraspinatus muscles, in combination with ESWT, compared to ESWT alone. In their randomized controlled trial involving 50 subjects, participants were divided into two groups. The kinesiotape, which was cut in a V‐shape, was applied 5 cm below the humeral insertion point for the deltoid muscle and from beneath the spine of the scapula to the supraspinous fossa, surrounding the upper corner of the scapula for the supraspinatus muscle. Additionally, the authors administered three sessions per week over three consecutive weeks of ESWT at 4 Hz, 1800 pulses, and an EFD ranging from 0.07 to 0.15 mJ/mm^2^ without local anesthesia. The control group underwent only the ESWT protocol. Frassanito and colleagues concluded that ESWT+KT showed a significantly greater improvement than ESWT on VAS.

### 3.4 Disodium EDTA

Cacchio et al. [21] conducted a randomized controlled trial (RCT) to evaluate the effectiveness of disodium EDTA combined with ESWT in treating RCCT, leveraging its ability to bind and help remove calcium deposits. The study group received a single‐needle mesotherapy with disodium EDTA, procaine, and injectable water once a week for 3 weeks, along with 15 min of pulsed‐mode US (frequency 1 MHz, power 2.5 watts/cm^2^, pulse mode 1:4), using a 15% disodium EDTA gel solution applied five times a week for three weeks. The control group underwent a sham treatment (the ultrasonic generator was not turned on, and disodium EDTA was not included in the mesotherapy mixture or the ultrasonography gel). 1 week after treatment and at the 1‐year follow‐up, the study group showed statistically significant improvements in mean total scores (*p* < 0.01) and individual item scores (*p* < 0.01 for all) in the CMS; no significant difference was observed in the control group regarding the CMS total score (*p* = 0.69). Additionally, a significant reduction in VAS scores was seen 1 week after treatment in the study group (*p* < 0.01), while no change was detected in the control group (*p* = 0.08).

### 3.5 PRP

Oudelaar and colleagues [22] conducted a single‐center, double‐blinded randomized controlled trial involving 80 patients who were assigned to receive either aspiration combined with corticosteroid injection or aspiration with PRP injection. The study aimed to evaluate pain, shoulder function, and quality of life at baseline and at 6 weeks, 3, 6, 12, and 24 months after treatment. Clinically significant improvements favoring the aspiration plus PRP group were observed only at the 6‐month follow‐up for NRS and CMS scores. In contrast, notable clinical differences favoring the aspiration plus corticosteroids were seen at the 6‐week follow‐up across all clinical scores except the NRS. The authors concluded that the combination of aspiration and corticosteroid injection provides an early beneficial effect on pain and function, suggesting that this approach should remain the treatment of choice for patients with RCCT.

### 3.6 Needling

In 2005, Krasny and colleagues [28] evaluated the effectiveness of US‐guided needling followed by high‐energy ESWT compared to ESWT therapy alone. The study group received an 18 G needle puncture repeatedly directed into the calcified deposit, using the lateral/longitudinal scan position for accurate guidance. After needling, they underwent ESWT, with 2500 impulses at an EFD of 0.36 mJ/mm^2^. Improvement in the CMS (*p* < 0.001) was observed in 54 (67.5%) of the 80 patients. Although the improvement was more pronounced in group I, no significant difference was reported between the two groups: 30 of 40 patients in group I and 24 of 40 in group II. Zhu et al. [29] analyzed 81 patients with RCCT treated with ultrasound‐guided needle puncture therapy. Forty‐one subjects in Group A received US‐guided percutaneous needle punctures and aspiration of deposits, while Group B received US‐guided punctures only. In Group A, VAS significantly decreased at 3 weeks post‐treatment; in Group B, VAS also significantly decreased, with efficacy detectable as early as 2 weeks post‐treatment. They concluded that needling calcific deposits without aspiration is an effective treatment for RCCT, indicating that removing calcified deposits does not affect patient outcomes. Kim and colleagues [30] compared US‐guided needling with subacromial corticosteroid injection and ESWT for restoring function and relieving pain. Both groups showed significant improvement in VAS, but the ESWT group experienced slight pain worsening 12 months after treatment compared to the US‐guided needling group. In the RCT by Louwerens et al. [27], 82 patients were randomly assigned to receive ESWT (2000 pulses at an EFD of 0.35 mJ/mm2) in four sessions with one‐week intervals, or US‐guided needling combined with corticosteroid US‐guided subacromial bursa injection. They stated that both techniques effectively improved function and pain with high satisfaction rates after 1 year of follow‐up; however, US‐guided needling was more effective in eliminating the calcific deposit, with higher additional treatments in the ESWT group.

### 3.7 US‐Guided Aspiration

de Witte and colleagues [61] assigned 48 patients with RCCT to two groups. The treatment group received US‐guided needling and aspiration combined with a US‐guided corticosteroid injection in the subacromial bursa, while the control group was treated with only a US‐guided corticosteroid injection in the same area. At 1‐year follow‐up, the mean CMS in group 1 was 86.0 (95% CI, 80.3–91.6) versus 73.9 (95% CI, 67.7–80.1) in group 2 (*p* = 0.005). They concluded that while both groups showed improvements, the results were significantly better in the aspiration group. In 2017, De Boer et al. [33] hypothesized that US‐guided needling would lead to a greater reduction in calcific deposit size and better clinical outcomes compared to ESWT. In the needling group, patients were treated with a single puncture using two hollow 18‐gauge needles to flush saline through both needle portals to wash out the calcium; in the ESWT group, patients underwent four sessions with 500 pulses at 1.5 bar and a frequency of 4.5 Hz, followed by 2000 pulses at 2.5 bar and a frequency of 10 Hz (EFD 0.10 mJ/mm). At 6 weeks, no significant difference in NRS (*p* = 0.15) was observed between the needling and ESWT groups. However, a significant interaction was found for CMS (*p* = 0.020), indicating that the needling group improved more than the ESWT group.

### 3.8 ESWT

Several studies have analyzed the effectiveness of ESWT in RCCT. To categorize the results, we divided the treatments into high (EFD > 0.3 mJ/mm^2^), medium (EFD > 0.2 mJ/mm^2^ but < 0.3 mJ/mm^2^), and low (EFD < 0.2 mJ/mm^2^) EFD groups. Perlick et al. [38], in an experimental and clinical study, examined two groups of 40 patients each who received 2000 impulses twice at EFD levels of 0.23 mJ/mm^2^ or 0.42 mJ/mm^2^. After 1 year, the CMS increased from 46 to 68 at 0.23 mJ/mm^2^ and from 48 to 73 points at 0.42 mJ/mm^2^. Pleiner and colleagues [39] compared two different ESWT regimens. Thirty‐one shoulders were treated with 2 × 2000 impulses of 0.28 mJ/mm^2^ at two‐week intervals (treatment group), and 26 shoulders received 2 × 2000 impulses of less than 0.07 mJ/mm^2^ at two‐week intervals (control group). Improvement in CMS was significantly higher in the treatment group at all follow‐up visits (*p* < 0.05). Regarding pain reduction, there was a significant improvement in the treatment group compared to the control group at the one‐week follow‐up (*p* < 0.05). However, no significant difference in pain was observed at the 3‐ and 7‐month visits. Albert and colleagues [40], in a prospective randomized trial of RCCT, compared the efficacy of two treatment sessions delivering 2500 extracorporeal shock waves at either high or low energy. At a mean of 110 days (range 41 to 255 days) after treatment, the increase in CMS was significantly greater (*p* = 0.026) in the high‐energy group than in the low‐energy group. The baseline improvement was significant in the high‐energy group, with a mean gain of 12.5 points (range, −20.7 to 47.5) (*p* < 0.0001). The improvement was less substantial in the low‐energy group. et al. [41] also conducted a prospective, randomized, observer‐blind study involving 50 patients divided into two groups, receiving navigated and X‐ray‐assisted ESWT at weekly intervals. Specifically, group I underwent three sessions of low‐energy treatment (0.08 mJ/mm^2^; 1000 impulses) without local anesthesia, while group II received two sessions of medium‐energy treatment (0.2 mJ/mm^2^; 2000 impulses) with subacromial anesthesia. Both groups showed significant clinical improvement (*p* < 0.0001) in CMS and VAS scores; however, there were no significant differences between the groups. Similarly, Farr and colleagues [42], 4 years later, compared a single high‐level ESWT (0.3 mJ/mm^2^) with a low‐level ESWT applied twice at weekly intervals (0.2 mJ/mm^2^). Both groups experienced significant pain reduction during movement and functional improvement (smd = 0.2, sd = 3.5 vs. smd = −0.3, sd = 3.5). Conversely, no significant pain reduction at rest was reported. The Italian group of Ioppolo and colleagues [43] assessed ESWT effectiveness in 46 patients with RCCT, randomly divided into two groups receiving ESWT at EFD levels of 0.20 mJ/mm^2^ (group A) and 0.10 mJ/mm2 (group B). The results showed a significant clinical improvement based on mean CMS after 6 months in group A (*X* = 79.43, SD = 10.33) compared with group B (*X* = 57.91, SD = 6.53), indicating that higher EFD was more effective for pain relief and functional enhancement. In Kim and Kwak's study [62], all participants received nonsteroidal anti‐inflammatory drugs for 6 weeks and conventional physical therapy (20‐min hot pack, 15‐min transcutaneous electrical stimulation, 5‐min ultrasonography at 1 W/cm^2^), three times per week for 12 weeks. The treatment group also received ESWT with an EFD of 0.14 mJ/mm^2^, a frequency of 4 Hz or 240 impulses per minute, three times a week until 6 weeks, but not from 6 to 12 weeks. The CMS showed a significant group interaction (*p* < 0.05); additionally, the treatment group showed a greater, statistically significant decrease in pain at 2, 6, and 12 weeks compared to the control group (*p* < 0.05). Duymaz and Sindel [44] similarly investigated radial ESWT (rESWT) in relieving pain and improving function in RCCT alongside conventional physiotherapy. They studied 80 patients randomly divided into an rESWT group (*n* = 40), treated with physiotherapy plus rESWT once weekly for 4 weeks, and a control group (*n* = 40), treated only with physiotherapy including ultrasound, transcutaneous electrical nerve stimulation, shoulder stretching exercises, and ice applications. All subjects received 20 treatments, 5 days a week for 4 weeks. Patients in the rESWT group experienced a statistically significant reduction in pain scores (*p* < 0.001), demonstrating that rESWT is an effective, noninvasive method for pain reduction in RCCT. Finally, in 2022, Fatima and colleagues [45] conducted a parallel‐group, randomized trial where the study group received ESWT plus conventional rehabilitation—12 sessions of 2000 shockwaves at 0.32 mJ/mm^2^ over 6 weeks (two sessions per week). The control group received only conventional physical therapy. No significant difference was observed in pain and CMS scores between the groups at baseline, or at 6 and 12 weeks after treatment (*p* < 0.05). Within‐group analysis also showed statistically significant improvements after treatment (*p* < 0.05).

### 3.9 Network Meta‐Analysis

**FIGURE 2:**
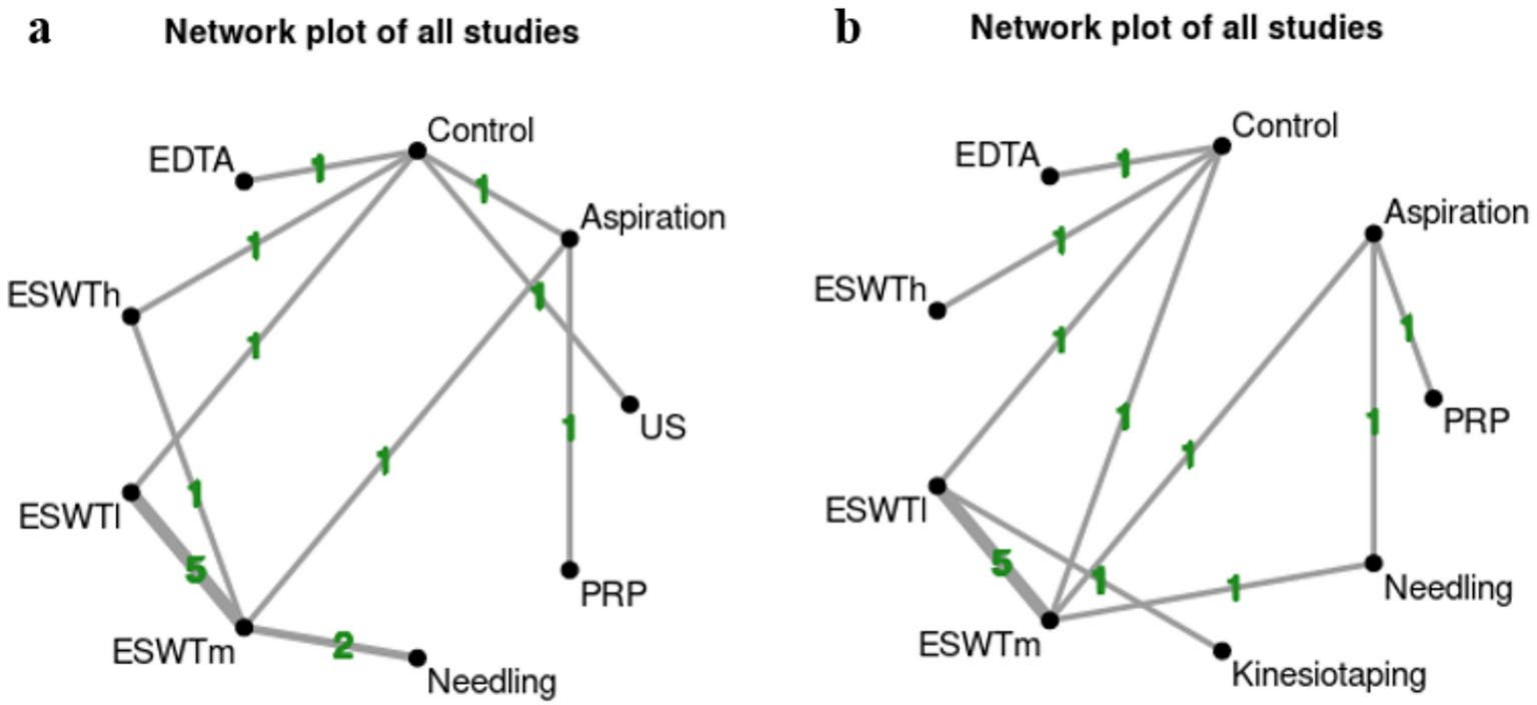
Network plots illustrating the direct and indirect comparisons among conservative treatments for rotator cuff calcific tendinopathy. (a) Network of interventions based on the Constant–Murley Score (CMS) for functional improvement. (b) Network of interventions based on pain reduction. Each node represents a treatment modality, with node size proportional to the number of patients and edge thickness reflecting the number of direct comparisons between treatments. EDTA: Ethylene diamine tetra‐acetic acid; ESWTh: High‐energy extracorporeal shockwave therapy; ESWTm: Medium‐energy extracorporeal shockwave therapy; ESWTl: Low‐energy extracorporeal shockwave therapy; PRP: Platelet‐rich plasma; US: Ultrasound.

This systematic review with network meta‐analysis aimed to thoroughly assess the role of various conservative treatments in patients affected by RCCT. When two interventions are connected, especially with an RCT, we can provide direct evidence comparing the two treatments; however, through a network of similar interventions, we can also analyze the combined effects (both direct and indirect), as shown in Figure 2.

**FIGURE 3:**
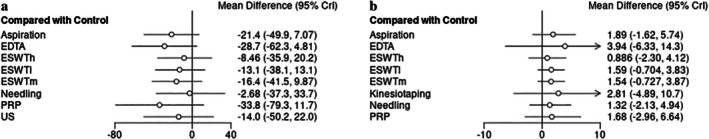
Pairwise forest plot illustrating the direct and indirect comparisons between interventions versus control using a network meta‐analysis approach (Constant–Murley score, a; pain b). EDTA: Ethylene diamine tetra‐acetic acid; ESWTh: Extracorporeal shockwave therapy high; ESWTl: Extracorporeal shockwave therapy low; ESWTm: Extracorporeal shockwave therapy medium; PRP: Platelet‐rich plasma; US: Ultrasound.

Regarding CMS, we evaluated 9 interventions on 895 patients in the network, 15 studies for a total possible 36 pairwise comparisons. Moreover, we evaluated 9 interventions on 770 subjects included in the network, 14 trials for a total possible 36 pairwise comparisons in perceived pain. Backward, we considered the group not subjected to the intervention as the control reference of the network, comparing each intervention included by reformulating a pairwise forest plot, as described in Figure 3.

**FIGURE 4:**
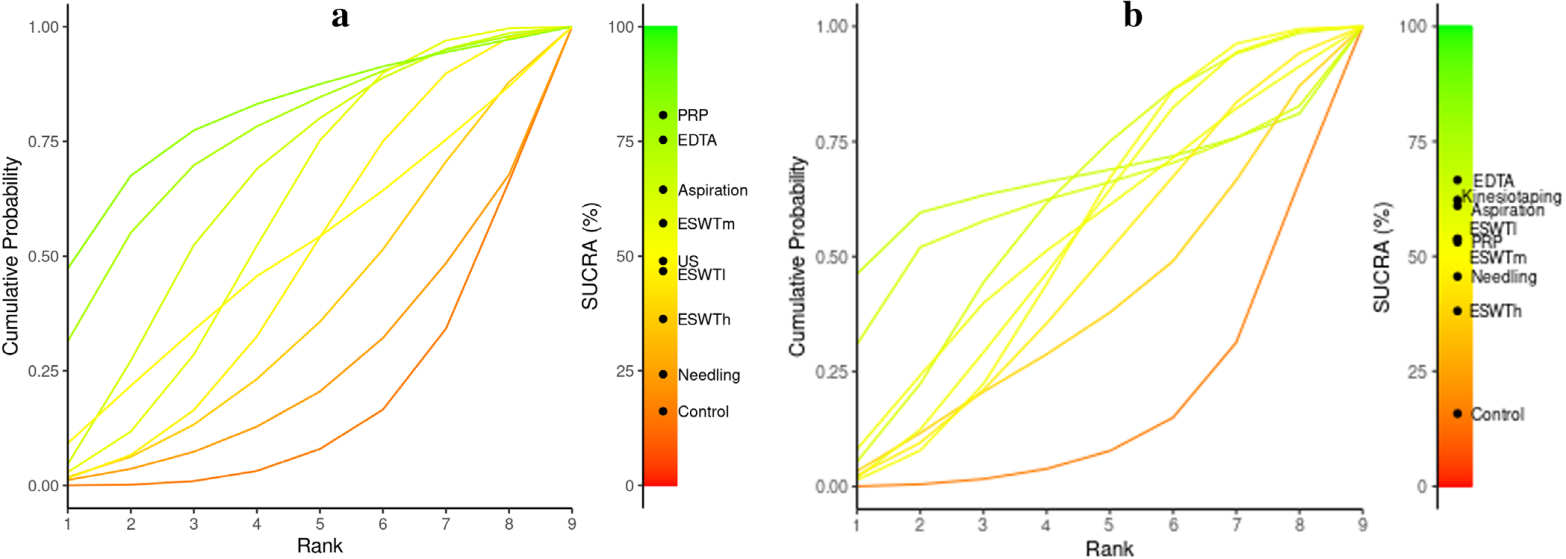
Surface under the cumulative ranking curve (SUCRA) plots for all interventions analyzed in the network meta‐analysis. (a) SUCRA rankings based on functional recovery measured by the Constant–Murley score (CMS). (b): SUCRA rankings based on pain relief. Higher SUCRA percentages indicate a greater probability that an intervention is among the most effective for the given outcome. EDTA: Ethylene diamine tetra‐acetic acid; ESWTh: High‐energy extracorporeal shockwave therapy; ESWTm: Medium‐energy extracorporeal shockwave therapy; ESWTl: Low‐energy extracorporeal shockwave therapy; PRP: Platelet‐rich plasma; US: Ultrasound.

Therefore, we created a network league table to rank all interventions, with treatments arranged from best to worst along the leading diagonal. The included studies presented comparable populations (adult patients with RCCT), similar outcome measures (primarily VAS/NRS for pain and CMS for function), and interventions applied in comparable clinical contexts. Estimates from pairwise meta‐analyses were positioned above the leading diagonal, while estimates from network meta‐analyses were located below it, as illustrated in Figure 4 for the CMS scale and for the pain score. Additionally, we conducted a SUCRA analysis for CMS recovery and pain relief scores to indirectly identify the best therapeutic option for RCCT. Based on the CMS analysis, PRP exhibited the highest probability of being the best choice available (85% probability), followed by EDTA (75%), aspiration (65%), and ESWTm (57%). In terms of perceived pain scores, EDTA scored 66%, kinesiotaping 62%, and aspiration 61%, while both ESWTl, ESWTm, and PRP had a probability of 53%, as depicted in Figure 4.

### 3.10 Quality Assessment and Risk of Bias

**FIGURE 5:**
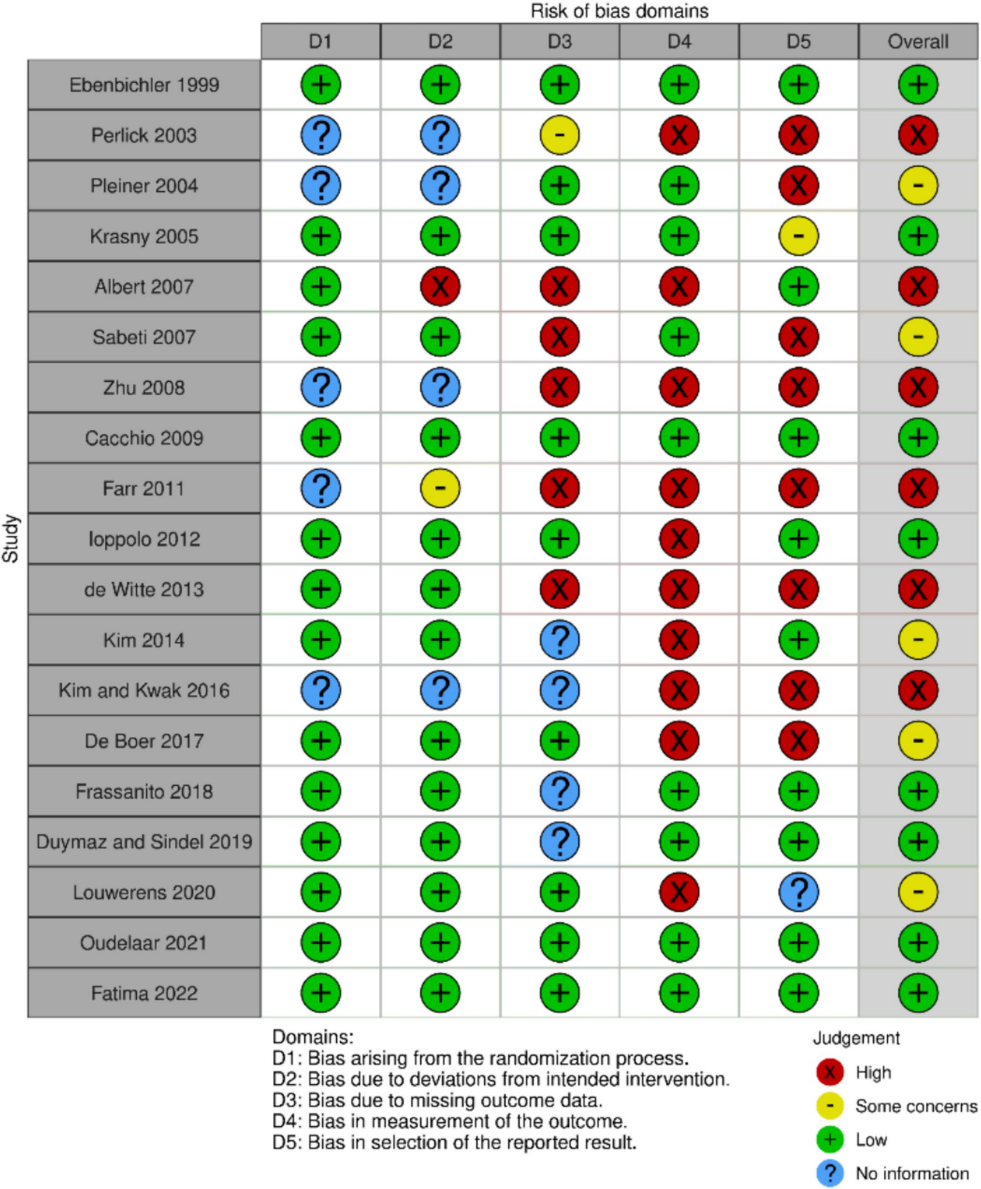
Risk of bias domains of included RCTs (traffic light plot).

Using the Cochrane risk‐of‐bias tool Rob2 [56], we evaluated the quality of the studies and found that seven RCTs (36,8%) [18, 21, 22, 28, 43, 44, 45, 60] were classified as high‐quality studies with a low risk of bias. In contrast, six RCTs (21.05%) [29, 38, 40, 42, 61, 62] were categorized as low‐quality studies with a high risk of bias. Five studies [27, 30, 33, 39, 41] expressed some concerns. Some studies lack data on randomization, outcome reporting, and selection in the reported results, which affects the overall assessment [18, 27, 29, 30, 38, 39, 42, 44, 62]. The quality scores for each assessment criterion are detailed in Figure 5.

## 4 Discussion

This systematic review and network meta‐analysis aimed to identify the most effective conservative treatment for reducing pain and improving function in individuals with RCCT. The pairwise comparisons conducted through the network meta‐analysis showed a statistically significant random effect, indicating an overall reduction in pain and improvements in function following conservative interventions. Regarding shoulder function, PRP emerged as having the highest probability of being the best option (85%), followed by EDTA (75%), aspiration (65%), and ESWT at medium energy flux density (EFD) (57%). Additionally, for perceived pain scores, the results were 66% for EDTA, 62% for kinesiotaping, and 61% for aspiration; then 53% for both low and medium EFD ESWT and PRP.

### 4.1 Comparison of the Efficacy of Conservative Treatments

As previously mentioned, many authors have proposed various conservative approaches for treating RCCT, especially considering that the condition often resolves spontaneously. However, while episodes of acute pain may reflect the initial resorption of calcific deposits, the timing of intervention remains crucial. Several factors appear to influence the response to conservative treatment, which can help customize therapy. These include the presence of bilateral deposits, calcification extending medially beyond the acromioclavicular joint, and the location in the anterior part of the acromion [63]. Prognostic factors significantly decrease the likelihood of failure with nonoperative therapy [64]. Conversely, Gartner type III calcific deposits and the absence of sonographic sound extinction on ultrasound imaging seem to indicate favorable outcomes [65]. Our findings support the efficacy of interventions in improving both pain and function in RCCT. The study by Cacchio et al. [21] concluded that EDTA is more effective than sham treatment in reducing VAS scores and improving CMS both in short‐ and long‐term follow‐up; however, EDTA was administered in combination with ultrasound (US), due to its anti‐inflammatory effects. Similarly, Oudelaar and colleagues [22] reported that PRP is more effective than corticosteroids in the long run, while corticosteroids showed better efficacy in the short term; in this randomized controlled trial, both approaches were combined with needle aspiration. Therefore, the outcomes were influenced by the combination of interventions. These combined protocols were considered during data synthesis and are explicitly noted as potential sources of bias. Furthermore, while SUCRA rankings identified EDTA among the most effective interventions, its isolated effect remains uncertain due to co‐interventions. Subacromial corticosteroid injections are commonly used in the conservative management of RCCT, especially during acute painful phases, but their role remains controversial. Some studies have reported short‐term benefits for pain relief and functional improvement, such as Oudelaar et al. [22], where corticosteroid injections combined with aspiration showed significant improvements at 6 weeks compared to PRP. Similarly, Kim et al. [30] and Louwerens et al. [27] included corticosteroids after needling procedures, emphasizing their role in multimodal protocols. However, other studies suggest corticosteroids may interfere with the natural resorption of calcifications, potentially delaying recovery or causing recurrence [66]. In our analysis, corticosteroid injections were rarely used alone and often served as adjuncts, limiting the ability to assess their independent therapeutic effect. For this reason, they were not ranked separately in the SUCRA model. Given the conflicting evidence, further randomized trials comparing corticosteroids alone or within standardized treatment protocols are needed to clarify their role in RCCT management, especially concerning timing, dosing, and long‐term results. Lavage and needling were first performed under fluoroscopy guidance by Comfort and Arafiles [67]; in 1995, Farin et al. [68] proposed using ultrasound (US) guidance for bursal lavage and needling. Currently, both needle aspiration and needling are considered safe and effective and are widely used clinically. However, even for these procedures, only two RCTs [29, 33] have tested aspiration and needling alone. Most protocols also include subacromial corticosteroid injections following the procedure. Finally, regarding ESWT, there is significant variation in EFD, number of impulses, and treatment intervals [69]. In general, several energy doses are used for RCCT treatment, with most authors reporting good outcomes with low‐ and medium‐energy ESWT [40, 43]. Ogon et al. defined failure of conservative therapy as persistent symptomatic tendinopathy after 6 months post‐treatment [64]. In such cases, surgical removal of the deposit via arthroscopy is the only option [70, 71]. Still, there is ongoing debate about whether to perform acromioplasty during this procedure and how to manage the rotator cuff defect after removing the calcific deposit [72].

### 4.2 Ranking of Interventions and Clinical Implications

The SUCRA‐based ranking allowed us to establish a hierarchy of interventions based on their probability of being the most effective. Platelet‐rich plasma (PRP) showed the highest probability (85%) of improving function, followed by EDTA (75%), aspiration techniques (65%), and ESWT (57%). For pain reduction, EDTA ranked highest (66%), followed by kinesiotaping and needle aspiration (both at 61%). However, the interpretation of rankings must consider the role of co‐interventions. For example, EDTA was never tested in isolation and was always paired with ultrasound. Similarly, PRP and corticosteroids were often administered after aspiration, limiting the assessment of their standalone effects. These combinations may inflate or mask the real effectiveness of single components. Despite these limitations, ranking interventions helps clinicians prioritize treatment options and optimize patient‐specific protocols. For instance, PRP may be favored in long‐term management strategies, while corticosteroids might be reserved for acute exacerbations in selected cases.

### 4.3 Recommendations for Conservative Management of RCCT

Based on current evidence, a multimodal, individualized approach appears most appropriate for managing RCCT conservatively. Clinicians should consider both patient characteristics and treatment availability when choosing among PRP, EDTA, aspiration, and ESWT. However, some treatments (especially corticosteroid injections) require further investigation. Although widely used, they present conflicting evidence and may need better‐defined indications. Likewise, standardized protocols for PRP preparation, ESWT parameters, and lavage/needling techniques are essential for future reproducibility and effectiveness. For patients not responding to conservative options after 6 months, as suggested by Ogon et al. [64], surgical intervention (e.g., arthroscopic removal) becomes the only viable solution. Yet even here, debate persists regarding the need for acromioplasty and the management of the rotator cuff defect [70, 71, 72].

### 4.4 Limitations and Future Directions

However, this systematic review with network meta‐analysis has some limitations, and the findings should be interpreted cautiously. First, we only included studies published in English, which could contribute to publication bias; in addition, although three recommended databases were used for the search, it is possible that other relevant articles could have been found by searching additional databases. Second, the treatments examined were diverse, not only in type but also in delivery parameters; for example, regarding ESWT, the included RCTs used different numbers of sessions and varying EFDs, and the stratification may not have fully addressed potential bias. Similarly, methods for PRP preparation and injection frequency were inconsistently reported. These inconsistencies may have affected the estimated treatment effects, creating clinical heterogeneity that might not be entirely mitigated by subgroup or sensitivity analyses. Although we attempted to categorize ESWT into low‐, medium‐, and high‐energy groups, variability remained within each subgroup, possibly impacting the internal consistency of the network meta‐analysis. Such variability can mask true effect sizes and limit the generalizability of the results. Third, there was heterogeneity regarding the duration from symptom onset to treatment. Fourth, the statistical method used allowed for indirect comparisons, meaning that some treatments were not directly compared within RCTs; moreover, these indirect comparisons are inherently limited by differences in study‐level factors such as baseline symptom severity, comorbidities (e.g., diabetes, thyroid dysfunction), and timing from symptom onset to treatment. These factors may threaten the transitivity assumption and introduce indirectness when evaluating treatment effects across different time points or multi‐component interventions. Fifth, some included RCTs had methodological limitations, such as a high or unclear risk of bias in domains like allocation concealment or blinding. These limitations reduce confidence in the effect estimates and may exaggerate the apparent efficacy of some treatments. Finally, we included studies with different outcome measures and follow‐up durations. We recognize that this approach might have excluded high‐quality studies using other validated tools, such as the American Shoulder and Elbow Surgeons or Disability of the Arm, Shoulder and Hand scores, limiting the applicability of our findings. Additionally, both functional outcomes and pain measures are likely influenced by the length of follow‐up, indicating that outcomes could vary among procedures if a specific follow‐up period were standardized. Nonetheless, this study aims to present an updated synthesis of current evidence on conservative treatments for RCCT, as the network meta‐analysis includes the largest number of RCTs on this topic to date.

## 5 Conclusion

Taken together, the findings of this systematic review with network meta‐analysis indicate that PRP or EDTA injections, ESWT, and needle aspiration could be considered promising and effective conservative approaches in reducing pain and improving shoulder function for managing RCCT patients. It is important to note that EDTA and PRP appear promising but require further validation through high‐quality RCTs that isolate their therapeutic effects. Given the limitations, our findings should be seen as a synthesis of the best available evidence so far rather than a definitive clinical guideline. More high‐quality, head‐to‐head RCTs are still necessary to address the risk of bias related to the heterogeneity of treatments discussed in this study and to consider pooling results across different scoring systems using standardized mean differences to establish a proper therapeutic algorithm for this disabling condition.

## Author Contributions

Conceptualization: L.M., M.M., and A.d.S. Methodology: L.M. and N.M. Software: G.L.B. and E.I.P. Validation: A.d.S. Formal analysis: L.M. Investigation: L.M. Resources: L.M. and N.M. Data curation: N.M. Writing – original draft preparation: L.M., N.M., and M.M. Writing – review and editing: L.M., M.M., G.G., and A.A. Visualization: M.M. and A.d.S. Supervision: G.G. and A.A. Project administration: M.M. All authors have read and agreed to the published version of the manuscript.

## Ethics Statement

The authors have nothing to report.

## Consent

The authors have nothing to report.

## Conflicts of Interest

The authors declare no conflicts of interest.

## Data Availability

The data that support the findings of this study are available from the corresponding author upon reasonable request.
